# Effect of oral zinc regimens on human hepatic copper content: a randomized intervention study

**DOI:** 10.1038/s41598-022-18872-8

**Published:** 2022-08-29

**Authors:** Ditte Emilie Munk, Tea Lund Laursen, Frederik Teicher Kirk, Hendrik Vilstrup, Aftab Ala, Lars Christian Gormsen, Peter Ott, Thomas Damgaard Sandahl

**Affiliations:** 1 Department of Hepatology and Gastroenterology, Aarhus University Hospital, Palle Juul-Jensens Boulevard 99, Aarhus N, Denmark; 2 Department of Nuclear Medicine and PET Centre, Aarhus University Hospital, Palle Juul-Jensens Boulevard 99, Aarhus, Denmark; 3 Institute of Liver Studies, King’s College Hospital NHS Foundation Trust, Denmark Hill, London, UK

**Keywords:** Liver diseases, Metabolic disorders

## Abstract

Zinc inhibits intestinal copper uptake, an effect utilized for treating Wilson’s disease (WD). We used copper-64 (^64^Cu) PET/CT to examine how much four weeks of treatment with different zinc regimens reduced the hepatic ^64^Cu content after oral ^64^Cu administration and test if alternative regimens were noninferior to the standard regimen of zinc acetate 50 mg × 3 daily. Forty healthy persons were randomized to four different zinc protocols. The WD standard treatment zinc acetate 50 mg × 3 reduced the hepatic ^64^Cu content from 26.9 ± 7.5% to 13.3 ± 5.6% of the administered ^64^Cu. Zinc gluconate 50 mg × 3 was noninferior (*P* = 0.02) (35.8 ± 9.0% to 17.4 ± 7.5%). Zinc acetate 150 mg × 1 (33.1 ± 9.9% to 17.4 ± 7.5%) and zinc gluconate 150 mg × 1 (28.1 ± 6.7% to 22.0 ± 6.7%) were less effective. These effects were intra- and inter-individually highly variable, and 14% had no effect of any zinc regimen, which may explain disparities in zinc treatment efficacy in WD patients.

## Introduction

Wilson’s disease (WD) is a rare autosomal recessively inherited disease caused by mutations in the *ATP7B* gene^1^*.* The protein product, ATP7B, mediates excretion of excess copper from liver to bile, which is crucial for whole-body copper control^2^. In WD, dysfunction of ATP7B results in copper accumulation in the body with subsequent organ damage, especially in the liver and brain^3^.

Treatment of WD initially aims to remove excess copper from the body, and after 1–2 years to maintain stable copper levels. The choice of WD treatment depends on disease severity and symptoms^4^. Most patients are initially treated with chelators that increase the amount of copper excreted into the urine. When the disease is stable, it may be preferable to change to zinc therapy owing to fewer and less serious side effects^5,6^. Many centers also use zinc as first-line treatment in asymptomatic WD patients identified by family screening.

The therapeutic effect of zinc is its inhibition of intestinal copper uptake. High doses of zinc inhibits intestinal copper uptake by inducing metallothionein (MT) synthesis in cells, especially in enterocytes^7–10^. This enhances the enteric copper binding and prevents intestinal copper uptake from food sources and intestinal juices, which will ultimately reduce the amount of copper presented to the liver and thus the hepatic copper content. Inhibition of intestinal copper absorption by zinc has been a recognized WD treatment option since the 1980’s, where a range of complex copper balance studies led to the current recommendation of three daily zinc doses all separated from meals^11–13^.

A specific concern with zinc treatment is reports of a higher rate of treatment failures compared to chelation therapy, while no specific risk factors for zinc therapy failure have been identified^14,15^. The use of zinc in WD is challenged by the dosing, three times daily separated from meals, which is difficult to adhere to for a life-long treatment. In addition, zinc acetate is the only zinc salt approved by the U.S. Food and Drug Administration (FDA) and The European Medicines Agency (EMA) for the treatment of WD. Due to unwanted effects and relatively high price, some patients prefer zinc gluconate or zinc sulphate. It is generally assumed that different zinc salts are equally effective but the evidence is limited^4,16^. Further insights into how dosing frequency and type of zinc salt affect zinc’s therapeutic effects would be of great value for informed decisions in WD.

The purpose of the present study was (1) to quantify the effect of four different zinc regimens (zinc acetate 50 mg × 3 daily, zinc acetate 150 mg × 1 daily, zinc gluconate 50 mg × 3 daily and zinc gluconate 150 mg × 1 daily) on hepatic copper content using ^64^Cu PET/CT, and (2) to compare the efficacy of these four regimens using a noninferior design. We hypothesized that the regimens had similar ability to inhibit copper uptake and the study was accordingly designed for noninferiority with zinc acetate 50 mg × 3 daily as the standard treatment. The study was carried out in healthy volunteers because the mechanism by which copper is absorbed from the gut is not affected by the WD mutation^17,18^.

We examined these questions by use of copper-64 (^64^Cu) PET/CT scans of the liver after oral administration of ^64^Cu. We have recently shown that ^64^Cu PET/CT can indeed quantify hepatic copper absorption and retention^18^. When administered orally, ^64^Cu is absorbed in the intestines and subsequently taken up predominantly in the liver with little copper distributed elsewhere^19^. In our previous studies, we demonstrated that the percentage of an orally administered dose of ^64^Cu that is recovered in the liver after 6 h remains relatively constant (34 ± 0.01%) in healthy persons^19^. For these reasons, a reduction of hepatic ^64^Cu content at a given time point reflects inhibition of intestinal absorption.

## Results

### Participants

A total of 38 out of 40 participants completed the trial. Non-completion was due to technical issues before the end-of-treatment scan in one participant and illness not related to the trial before the first scan in another participant. One participant was excluded from the analysis due to a technical error on the baseline scan, leaving 37 patients for the analyses.

There were no differences regarding sex, age, body mass index or baseline blood samples in the four groups (Table 1).

**Table 1:** Patient characteristics with selected blood samples.

	Zinc acetate 50 mg × 3 (n = 10)	Zinc acetate 150 mg × 1 (n = 9)	Zinc gluconate 50 mg × 3 (n = 9)	Zinc gluconate 150 mg × 1 (n = 9)	
**Sex**					
Male	4	4	4	1	
Female	6	5	5	8	NS
Age (years)	62.3 (8.2)	59.6 (18.6)	63.6 (9.4)	59.0 (5.8)	NS
Body mass index (kg/m^2^)	23.2 (3.3)	23.4 (2.5)	24.3 (2.3)	23.6 (3.1)	NS
Serum zinc (µmol/L)	11.3 (1.7)	12.3 (2.5)	11.0 (1.0)	11.4 (1.6)	NS
ALT^1^ (U/L)	25.2 (13.2)	27.8 (10.1)	20.7 (7.5)	24.7 (8.8)	NS
Bilirubin (µmol/L)	10.4 (2.8)	12.2 (6.0)	14.4 (4.7)	11.3 (5.7)	NS
Albumin (g/L)	41.2 (1.2)	41.2 (1.2)	40.7 (2.2)	42.2 (2.6)	NS
Creatinine (µmol/L)	69.6 (13.1)	61.9 (11.6)	65.9 (10.9)	61.1 (7.0)	NS

*NS* non-significant (*P* > 0.05).

^1^Alanine aminotransferase.

The mean (SD) tracer dose was 30.0 (2.2) megabecquerel (MBq) for the baseline scan, 30.9 (2.1) MBq for the end-of-treatment scan and 29.0 (2.8) for the extra baseline scan. The total radiation exposure for participants scanned twice was 3.9 (0.2) mSv and for participants scanned thrice 5.7 (0.7) mSv.

### Hepatic ^64^Cu content before and after treatment

The amount of ^64^Cu in the liver on the baseline scan was on average 31% (95% CI 28–33) of the administered dose (Table 2). As illustrated by the confidence interval, this fraction was quite constant like in previous studies^19^. This reflects that intestinal copper uptake is around 50% of the oral intake since at this time point (13 h after tracer intake), biliary copper excretion is also evident.

**Table 2:** Results.

	Zinc acetate 50 mg × 3 (n = 10)	Zinc acetate 150 mg × 1 (n = 9)	Zinc gluconate 50 mg × 3 (n = 9)	Zinc gluconate 150 mg × 1 (n = 9)	
Amount of hepatic copper-64 on end-of-treatment compared to baseline scan (%)	56 (37)	56 (33)	49 (21)	85 (40)	NS
Baseline SUV^1^	12.3 (3.2)	15.5 (4.5)	17.0 (2.4)	13.2 (2.9)	*P* = 0.02
End-of-treatment SUV^1^	6.2 (1.9)	8.2 (4.7)	8.2 (2.8)	10.4 (3.4)	NS
Baseline %AD^2^	26.9 (7.5)	33.1 (9.0)	35.8 (4.4)	28.1 (6.7)	*P* = 0.02
End-of-treatment %AD^2^	13.3 (5.6)	17.8 (10.3)	17.4 (7.5)	22.0 (6.7)	NS
Adherence violations	0	4	0	0	
Fasting violations	2	1	2	0	
**Adverse events possibly related to trial drug**					
Gastric discomfort	2	4	0	1	*P* = 0.001
Nausea	5	5	1	2	
Headache	0	2	0	0	
Palpitation	0	1	0	0	
Obstipation	0	2	0	0	
Total events	7	15	1	3	

	(n = 5)	(n = 5)	(n = 9)	(n = 4)	
**Serum zinc**^**3**^** (µmol/L)**					
Normal range 10–19 µmol/L	25.2 (5.0)	20.4 (4.0)	18.9 (4.3)	17.3 (1.5)	*P* = 0.04

*NS* non-significant (*P* > 0.05).

^1^Mean liver SUV.

^2^Percent of administered dose in the liver.

^3^After zinc administration.

**Figure 1:**
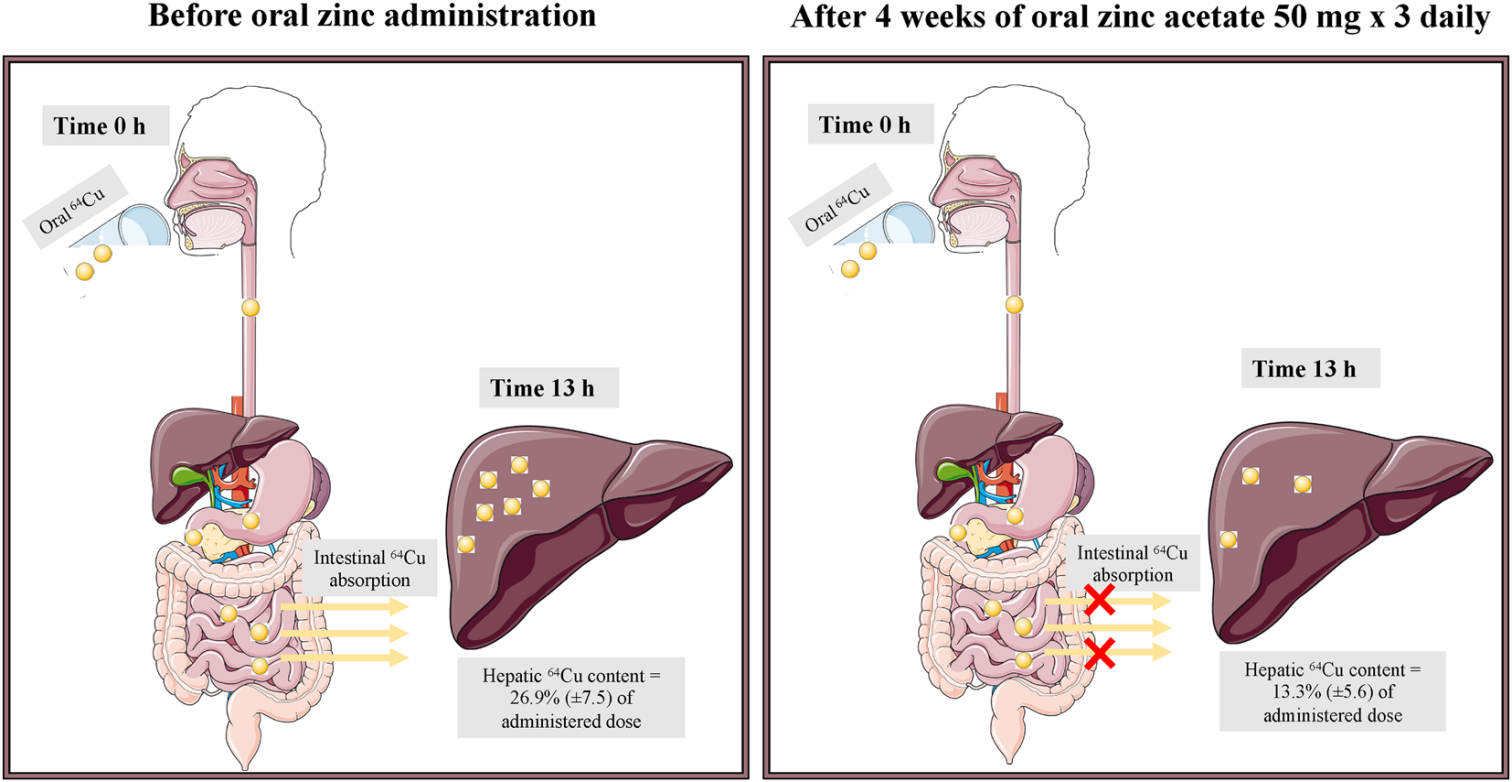
Visualization of method.

**Figure 2:**
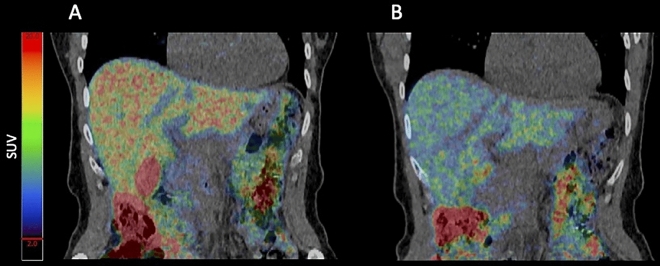
PET/CT scan 13 h after oral intake of 30 MBq copper-64. (**A**) Baseline scan. (**B**) End-of-treatment scan—after 4 weeks administration with zinc gluconate 50 mg × 3 daily.

At the end-of-treatment scan, this was reduced to on average 17% (95% CI 15–20) of the administered dose. Hence zinc administration approximately halved the hepatic copper content across all four groups (Figs. 1 and 2).

### Effect of the four different oral zinc regimens on hepatic ^64^Cu content

**Figure 3:**
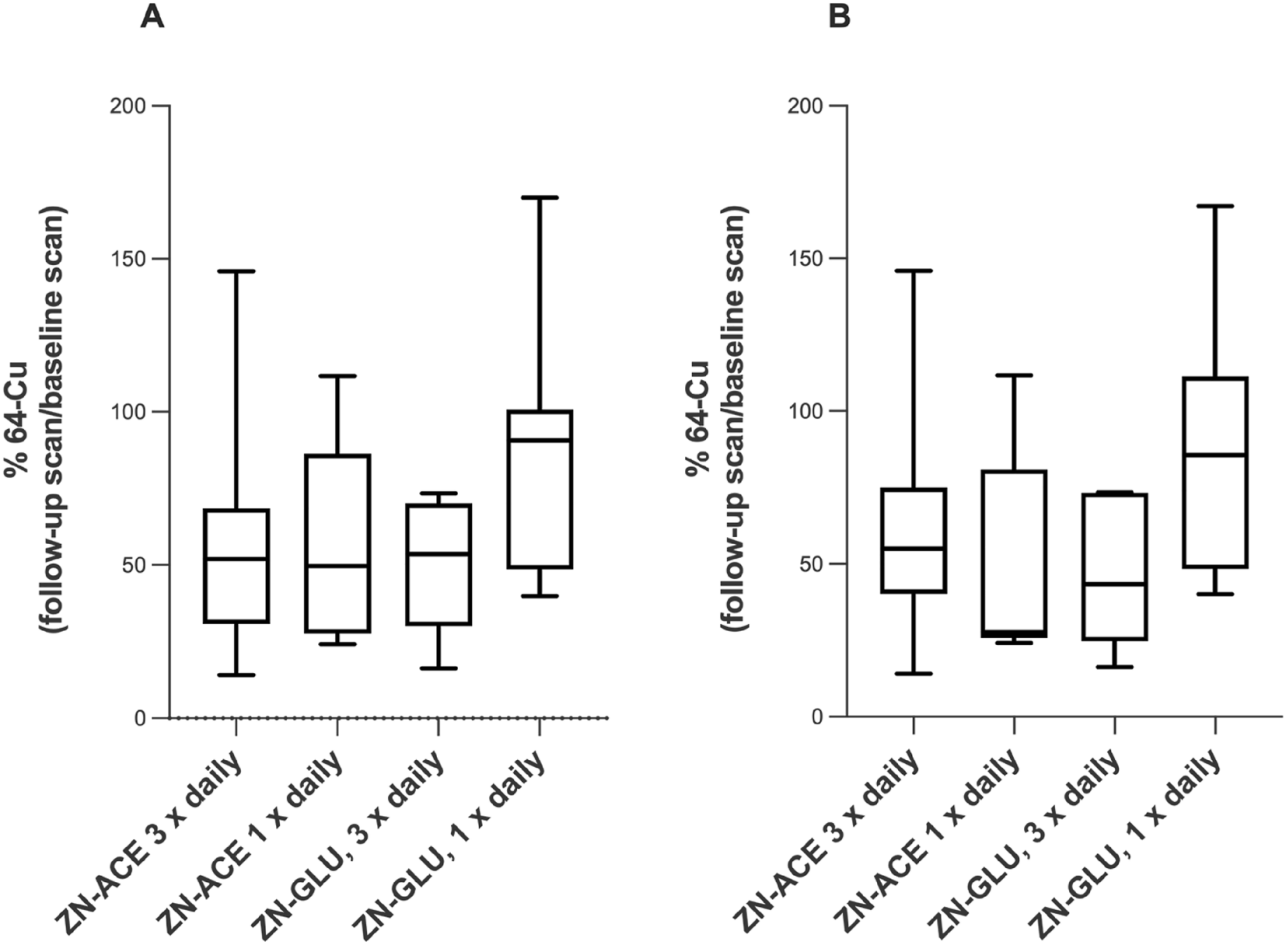
Box plot of hepatic copper-64 (mean hepatic SUV) as percentage on end-of-treatment scan compared to baseline scan. (**A**) All completed participants. (**B**) Per-protocol participants.

Figure 3 illustrates the treatment effects in each group. Zinc acetate × 3 daily (standard of care treatment) reduced the hepatic copper content to 56% (95% CI 29–82) of the baseline value, zinc acetate × 1 daily to 56% (95% CI 31–81) and zinc gluconate × 3 daily to 49% (95% CI 33–65) (Table 2, Fig. 3). These were all statistically significantly different from 100%, indicating the zinc-induced inhibition of intestinal absorption and hepatic uptake of ^64^Cu. Only in the zinc gluconate 150 mg × 1 group, the reduction to 85% (95% CI 54–116) of baseline value was not statistically lower than 100%, so there was no detectable effect of this regimen on intestinal absorption and hepatic copper content.

**Figure 4:**
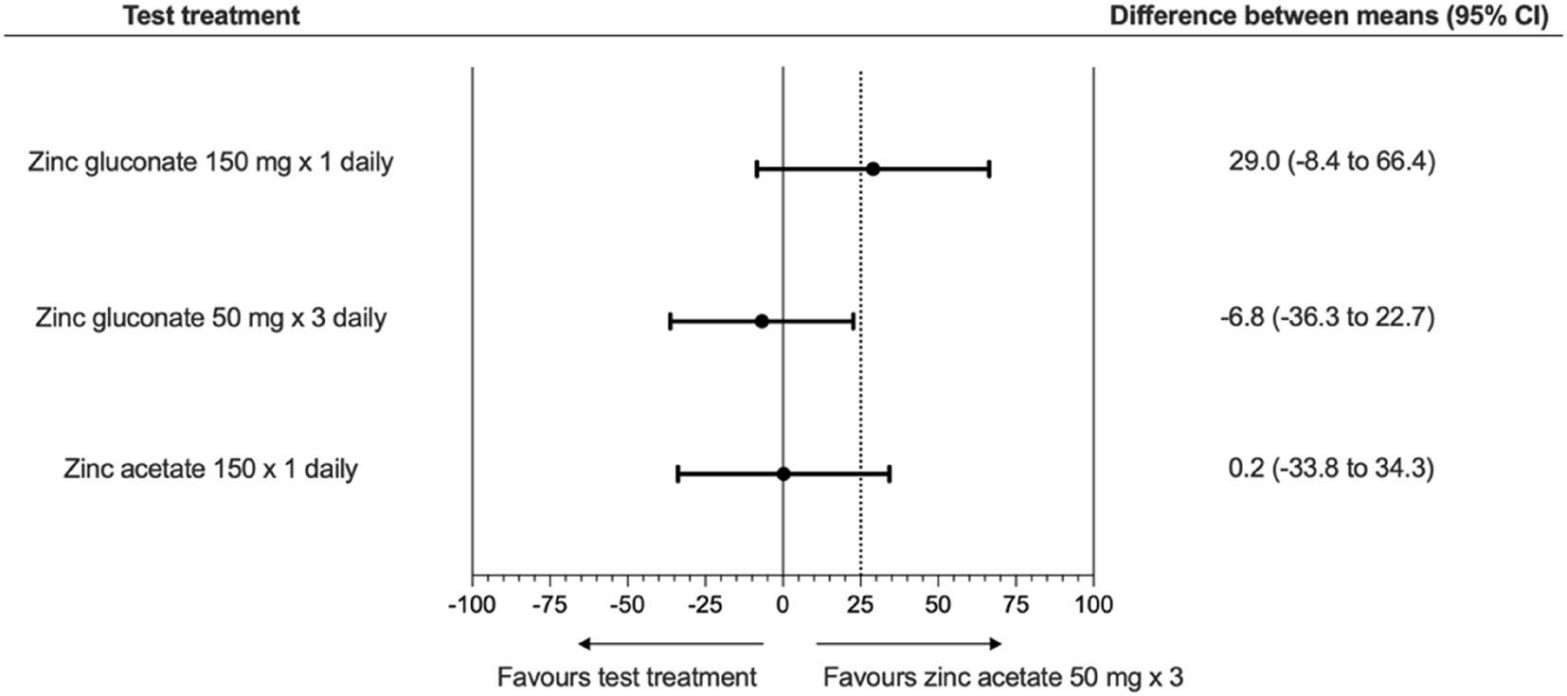
Illustration of the noninferiority analysis. Three regimens were tested for noninferiority against the standard regimen, zinc acetate 50 mg × 3. For each treatment, the difference between means (standard of care regimen vs. test regimens) for all completed participants is depicted with 95% confidence intervals. The dotted line represents the noninferiority margin, ∂. Because the 95% confidence limits of the zinc gluconate 50 mg × 3 regimen did not cross the noninferiority margin, this regimen was noninferior to the standard treatment. The two other regimens did not fulfil this criterion.

In the noninferiority analysis, only zinc gluconate × 3 daily was noninferior (but not superior) to zinc acetate × 3 daily (*P* = 0.02), while neither zinc acetate × 1 daily nor zinc gluconate × 1 daily fulfilled the criteria for noninferiority to zinc acetate × 3 daily (Fig. 4).

The hepatic copper content reductions were highly inter-individually variable and 14% (5/37) had no measurable effect of zinc (Table 2, Fig. 3).

### Intraindividual day-to-day variation

**Figure 5:**
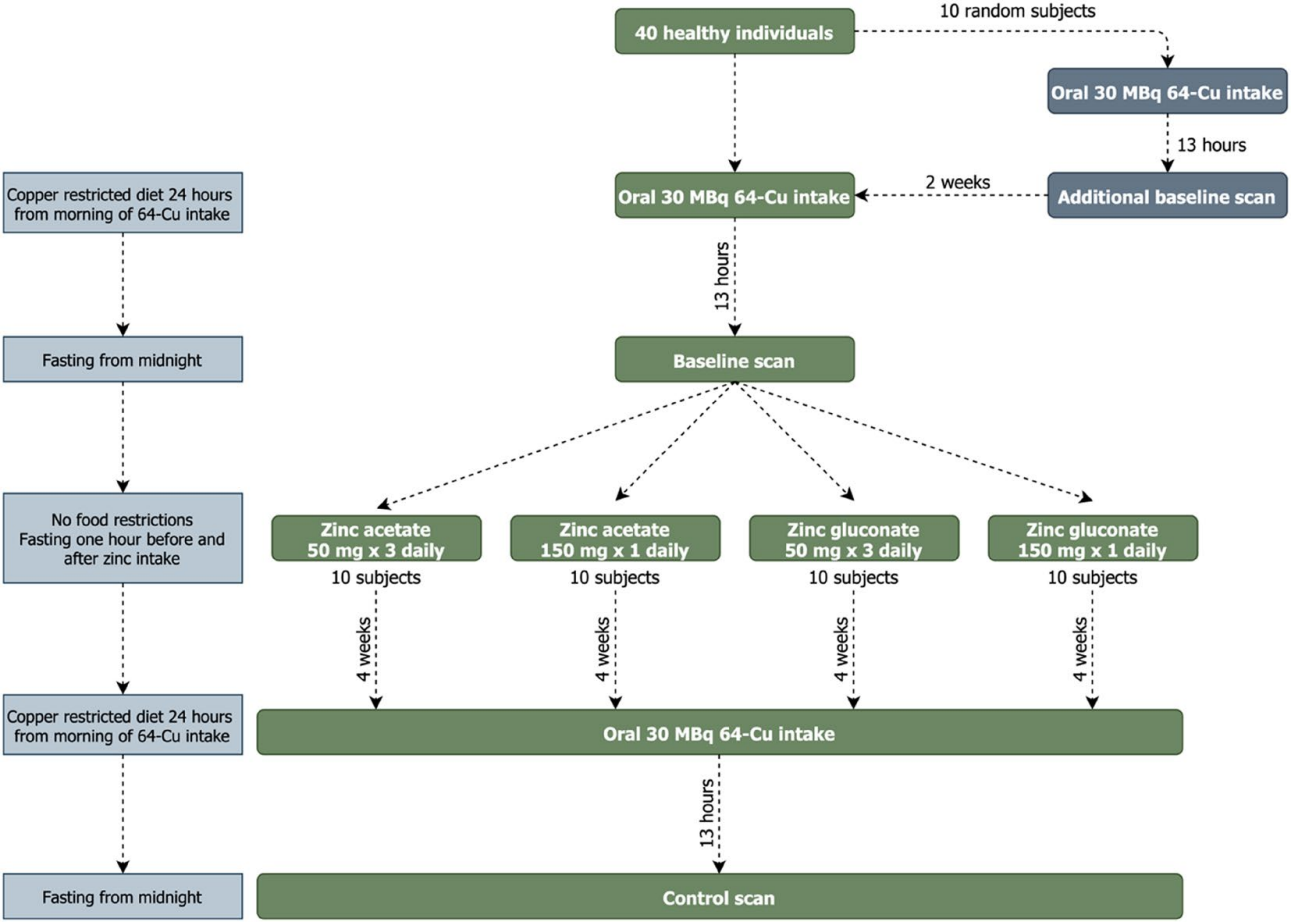
Study design.

In 10 participants with repeated baseline measurements (Fig. 5), the coefficient of variation (CV%) of the repeatedly measured hepatic copper content was 19.6% (95% CI 10.9–25.5). Hence with 10 participants, the method can determine a group difference in hepatic mean standard uptake value (SUV) of 25.5% or more, as a smaller difference may be due to day-to-day variation. Based on the Bland–Altman plot, variation did not depend on mean values (Supplementary materials, Figure S2).

### Serum zinc levels

The serum zinc levels after treatment were lower in the groups treated with zinc gluconate than in the groups treated with zinc acetate (*P* = 0.03 for 50 mg × 3 and *P* = 0.02 for 150 mg × 1 vs. standard of care treatment) (Table 2). These differences did not correlate with the change in SUV (r = 0.04, *P* = 0.9) or compliance (r = 0.31, *P* = 0.15) (Figure S4, supplementary material). Zinc concentrations in the groups treated with zinc gluconate remained below the upper normal level (< 19 µmole/L) (Table 2).

### Unwanted effects

At least one of the common side effects nausea, gastric discomfort, and headache occurred in 21/37 participants, thus a higher frequency than the usual 10%. Zinc acetate had more unwanted effects than zinc gluconate (*P* = 0.001, Table 2).

In total, nine protocol violations were encountered: Four participants had compliance below 90% (87%, 86%, 76% and 46%), and 5 participants did not comply with fasting rules (< 90%) (Table 2). Non-adherence in three participants was related to side effects in the zinc acetate 150 mg × 1 group, primarily gastric discomfort, and nausea. Non-compliance regarding fasting in two participants was related to side effects in the zinc acetate 50 mg × 3 group, primarily morning nausea and gastric discomfort.

Nausea and gastric discomfort were not related to the reduction of copper content. Excluding the inflicted participants from analyses did not alter the outcome (Supplementary material, Figure S3).

## Discussion

In this randomized, non-blinded intervention study involving healthy persons, 28 days of different oral zinc regimens approximately halved hepatic ^64^Cu content as determined by ^64^Cu PET/CT. This zinc-induced reduction in hepatic ^64^Cu content reflects zinc’s inhibition of intestinal ^64^Cu absorption as ^64^Cu is predominantly taken up in the liver after oral administration^19^. Three of the regimens (zinc acetate 50 mg × 3, zinc acetate 150 mg × 1 and zinc gluconate 50 mg × 3) reduced the hepatic copper content to a similar extent, but only zinc gluconate 50 mg × 3 was noninferior to the WD standard of care treatment zinc acetate 50 mg × 3. The fourth regimen, zinc gluconate 150 mg × 1, did not reduce the hepatic copper content. Five participants (14%) had no measurable effect of zinc (1 on zinc acetate 50 mg × 3, 2 on zinc acetate 150 mg × 1, 2 on zinc gluconate 150 mg × 1).

Our data in healthy persons suggest that zinc acetate and zinc gluconate are similar in their ability to reduce copper absorption if dosed three times daily. This observation is in line with a recent study comparing serum ALT and urinary copper levels in WD patients on different zinc preparations^20^. The assumption that the dose of elementary zinc is more important than the specific zinc salt is also supported by the fact that the first zinc treatment used was zinc sulphate^21,22^. To confirm that zinc gluconate is as effective as standard-of-care treatment, ideally prospective studies with clinical endpoints would be needed, but they are difficult to perform in such a rare disease, and more likely, data from retrospective databases may be used to assess long-term efficacy^23^.

Zinc gluconate dosed once daily did not lower the hepatic copper content. Zinc acetate dosed once daily reduced the copper content numerically to the same extent as did zinc acetate administered three times a day. However, the latter regimen exceeded the noninferiority margin, which implies a risk that it is less effective than the standard of care treatment. The finding is supported by a range of complex copper balance studies performed by G.J. Brewer in the 1980’s in 5, 14 and 11 WD patients, respectively^11–13^. Whilst these studies concluded that one daily dose of zinc therapy was less effective than two or three daily doses, they were based on a trend since the studies were underpowered^13^. Brewer’s team further tested if orally administered ^64^Cu and subsequent radioactivity counts in venous blood samples could quantify zinc’s effect on intestinal copper uptake^13,17^. The reduction in peak copper uptake after six weeks zinc treatment was roughly 90%^17^. This was far more than ≈ 50% in our study. However, only 0.2–10% of the administered ^64^Cu is present in the blood sampled 30–180 min after copper intake^17^. Moreover, when combining ^64^Cu administration with PET technology, we see that most of orally administered copper is rapidly taken up by the liver, remains in the gut lumen, or temporarily adheres to other organs, demonstrating that only measuring the peak serum concentration overestimates the effect of zinc^19^.

The day-to-day variation in baseline SUV (CV%: 19.6%) was relatively higher than what we expected. It is most likely due to a variation in intestinal copper absorption, arising from bowel movements and variation in food intake, especially of copper and zinc. We attempted to minimize such variation by having participants on a low-copper diet for 24 h, on an overnight fast, and by monitoring baseline zinc values. However, different diets are also thought to affect copper uptake, i.e., vegetarian food sources may have lower copper availability than meat products, and phytates, ascorbic acid and fiber may complex with copper reducing the uptake^8,24^. Further, a recent review suggested that dietary copper restrictions may have little impact on overall copper uptake^25^. Taking this into consideration, a controlled homogenous diet may be recommended for future studies to decrease the day-to-day variation.

As for now, the high day-to-day variation does not allow for individual zinc therapy guidance based on PET/CT evaluation, possibly except for those persons without measurable zinc effect.

Even with its limited sample size, our study illustrates that unwanted effects may be a problem with zinc therapy. Twenty-one out of 37 participants had a least one of the common side effects gastric discomfort, nausea, or headache. In general, zinc gluconate seemed more tolerable than zinc acetate. While we cannot rule out a lack-of-blinding bias, the observations are similar to reported clinical experience^20^. Two participants in the zinc acetate 50 mg × 3 group did not comply with fasting but nonetheless had large reductions in copper content (reductions to 28% and 34% of baseline values, group mean 56%). Thus, fasting may not be as important as it is currently considered.

While both 50 mg × 3 regimens (zinc acetate and zinc gluconate) were effective in reducing copper content, there was considerable variation, and even in the standard of care group, one participant had no measurable effect of zinc. Across all participants, lack of efficacy could not be related to adherence or side effects. Furthermore, we saw no relation between effect of regimen and serum zinc levels neither pre- nor post-treatment. We thus have no data to explain why some persons did not have any effect, but the finding may be related to the clinical observation that some WD patients are zinc treatment non-responders. In a long-term follow-up study, 14/88 patients on zinc therapy developed hepatic failure compared to 4/313 on chelation therapy (hazard ratio = 3.8) with no differences regarding serum and urinary zinc^15^.

Zinc adherence in WD patients is currently examined by serum zinc. As we saw no relation between serum zinc and regimen adherence or effect on copper uptake, this may be important for the management of WD patients, and better measures of adherence and effect are warranted. We did however not measure urinary zinc excretion, which may be a better measure of adherence, although its correlation to inhibition of intestinal copper absorption has not been examined^20^. In future studies, MT measured in leukocytes may be a better measure of zinc status^8,26,27^.

It is a limitation for the interpretation of our study that we did not directly measure intestinal copper absorption, on which zinc is recognized to exert its effect in WD. However, the direct disease-modifying effect of zinc is to decrease the presentation of absorbed copper to the liver and other organs. Therefore, we are confident that our measurements of hepatic copper content reflect the central pharmacodynamic mechanisms of zinc treatment in WD.

In general, zinc is a safe treatment with relatively harmless side effects, and our study suggests that if WD patients have side effects on standard treatment, it may be safe to change to zinc gluconate 50 mg × 3 daily. This finding has the potential to improve therapy adherence and thereby patient outcome. That said, whatever zinc treatment used, a few patients remain at risk of severe clinical deterioration, and current efficacy evaluation parameters cannot identify these patients. With further development and understanding of the day-to-day variation, the ^64^Cu PET/CT technique may be used to identify zinc non-responsiveness earlier, rather than by awaiting untoward clinical development.

In conclusion, our ^64^Cu PET/CT study showed that zinc therapy approximately halved intestinal copper absorption as determined by the reduction in hepatic SUV. Zinc gluconate 50 mg × 3 daily was noninferior to the current WD standard of care regimen zinc acetate 50 mg × 3 daily in its ability to reduce intestinal copper uptake. It is thus proposed as an alternative long-term zinc treatment. Once daily dosing was not supported due to lower effectiveness and for zinc acetate relation to many side effects. Moreover, we demonstrated that the effect of zinc on hepatic copper content was highly variable and in some, absent, which may explain some of the variation in zinc treatment efficacy in WD patients.

## Methods

### Study design

Figure 5 illustrates the study protocol. Participants were randomized to receive one of four different zinc regimens and scanned using ^64^Cu PET/CT before and after four weeks of treatment (Fig. 5). Four weeks were chosen as an appropriate study period because of earlier studies demonstrating an increase in MT levels after 4–5 days of zinc treatment^28^. Furthermore, when changing patients to zinc therapy, it is recommended by the manufacturer of Wilzin® (EMA-approved zinc acetate) to continue with other therapy for 2–3 weeks to ensure maximal MT induction, which is supported by another guideline^29^.

The scans were performed 13 h after oral administration of 30 MBq ^64^Cu. The time point was chosen based on earlier results which showed a nearly constant hepatic ^64^Cu content from 6 to 20 h after oral tracer administration^19^.

The zinc regimens were (1) zinc acetate (“Wilzin”, Recordati AB, Kista, Sweden) 50 mg × 3 daily, (2) zinc acetate 150 mg × 1 daily, (3) zinc gluconate (“Zink Mineral”, Natur-Drogeriet, Hørning, Denmark) 50 mg × 3 daily and (4) zinc gluconate 150 mg × 1 daily. Furthermore, 10 randomly selected persons were scanned twice without zinc administration to determine the intraindividual day-to-day variation in hepatic ^64^Cu content.

Participants were block-randomized to receive one of four treatments. The allocation sequence was made using a randomization website (www.randomization.com) which produced a list of randomization numbers and the associated treatment modality. As participants were included, they received an inclusion number which matched a randomization number. Participants were informed which treatment modality they had been randomized to when they received the tablets after the baseline scan. The randomization list could be assessed by investigators at any time.

Correct completion of the trial required intake of > 90% of the doses and fasting one hour before and after for > 90% of the zinc tablet administrations. Adherence is presented as percentage and was calculated by counting returned study tablets after the treatment period. Fasting was controlled using a logbook where the participants for each tablet intake recorded if they had fasted correctly or not.

Twenty-four hours before the scan, the participants adhered to a copper-restricted diet (avoiding shellfish and seaweed, liver, nuts and seeds, cocoa, olives, chickpeas, champignons, soy flour and sundried tomatoes) and 8 hours before the scan, they were asked to fast (water allowed).

### Participants

The clinical evaluation of participants was carried out at the Department of Hepatology and Gastroenterology, Aarhus University Hospital.

A total of 40 healthy participants were recruited from May 30, 2019, to January 28, 2020, by advertisements at the hospital, on a trial participant recruitment website and in a local newspaper.

All participants gave informed consent before entering the trial. The inclusion criteria were age > 50 years and body mass index < 30. The age criterium was a requirement from the Ethics Committee due to radiation exposure. Exclusion criteria were chronic liver or kidney disease, participation in a clinical trial including PET/CT within the past year, known hypersensitivity to copper or other ingredients in the tracer formula or claustrophobia. Unknown kidney or liver disease was excluded by an array of standard biochemistry (alanine transferase, alkaline phosphatase, international standardized ratio (INR), bilirubin, hemoglobin, platelets, albumin, c-reactive protein, estimated glomerular filtration rate, zinc, sodium, potassium, creatinine, carbamide).

### Blood samples

Blood samples were drawn at inclusion and immediately after the last scan and were analyzed by the Department of Clinical Biochemistry at Aarhus University Hospital, Denmark.

We added measurements of zinc levels halfway through the study to determine serum zinc levels after treatment. Consequently, it was not available in all participants.

Zinc levels were measured by direct colorimetric determination without deproteinization of zinc in serum samples. The normal range is 10–19 µmol/L.

### Radiochemistry

The ^64^Cu radioisotope was obtained from a commercial source (Hevesy Laboratory, DTU Nutech, Risø, Roskilde, Denmark) and subsequently the ^64^Cu tracer solution (a sterile acetate-buffered solution of ^64^CuCl_2_) was prepared and quality controlled at our center as previously described^19^. The tracer was administered orally approximately 13 h before the scan at a dose of 30 MBq in 5 mL of NaCl solution poured into a glass of water.

### ^64^Cu PET imaging

The PET/CT scans were performed at the Department of Nuclear Medicine and PET-center, Aarhus University Hospital. The ^64^Cu radioactivity in the liver was measured by a PET scan; further details on the procedure are described in Kjærgaard et al.^19^. An initial CT topogram was obtained to ensure that the liver, bile ducts and the proximal part of the gut were within the planned field-of-view. Furthermore, the low-dose CT scan was used for attenuation correction of the PET data and the definition of liver anatomy in the fused PET/CT images. PET images covering the area from the lower thorax to below the liver were acquired utilizing continuous bed motion with a scan speed of 0.4 mm/second allowing for good count statistics. The image reconstruction was iterative (UltraHD PET) with 4 iterations and 5 subsets, 2 mm Gaussian post filter and a 200 × 200 matrix.

### Outcome

To analyze the effect of treatment regimens, we calculated the change in mean SUV in the liver before and after treatment. The hepatic mean SUV is a measure of the hepatic ^64^Cu content at the time of the PET/CT scan, and the change in this measure reflects the reduction of hepatic copper content and thus the reduction of intestinal copper uptake induced by the treatment. Change in mean SUV from baseline to end-of-treatment was calculated as $\frac{mean\ SUV\ at\ baseline}{mean\ SUV\ at\ end-of-treatment}\cdot 100\%$. This relative measure was preferred to minimize interference from inter-individual variation in baseline tracer uptake.

Secondary outcomes were serum zinc levels, unwanted effects, and day-to-day variation in 10 subjects.

In a post-hoc analysis, we calculated the percentage of administered dose in the liver to supply a more intuitive measure of uptake than SUV.

### Image analysis

The scans were analyzed per protocol in PMOD (version 4.0, PMOD Technologies LLC). The amount of ^64^Cu in the liver was determined by the mean SUV in five spherical volumes of interest (VOI) with a diameter of 20 mm placed in the right liver lobe. This is equivalent to approximately 1.5% of the total liver volume. These spherical VOIs were chosen for SUV quantification instead of the entire liver due to signal spill-out from other organs (in this case especially colon and gallbladder) and respiratory motion. The positions of the VOIs on each participant’s first scan were determined by the shape of the liver in the transverse plane. The same positions on the second scan were ensured by their relation to the right kidney and by comparing the liver shape in the two scans. The amount of ^64^Cu in the gallbladder was determined using one VOI with a diameter of 10 mm in the center of the organ.

We calculated the percent of administered ^64^Cu dose in the entire liver as previously described using the radioactivity measured in kilobecquerel per milliliter (kBq/mL) in the VOIs^19^. This was done under the assumption that the uptake of ^64^Cu was homogenous, hence the VOI radioactivity would be the same in the entire liver, and further that the liver volume could be calculated with the validated formula^30,31^:

$$Liver\, volume = \left( {\sqrt {body\,surface\, area} \cdot 0.72 + 0.171} \right)^{3} \cdot 1000.{ }$$

### Sample size

The sample size calculation was based on a noninferiority design. The level of significance (α) was set to 0.05, and the statistical power (1 − β) was set to 0.90.

The study which underlies the current zinc dosing recommendations included 15, 4 and 6 persons in each intervention group, thus we imagined the group sizes somewhere in between^13^. In pilot studies, WD patients on zinc therapy had ≈ 80% lower hepatic SUV mean 6 h after oral tracer administration compared to healthy controls, even after 3 days treatment pause (unpublished data). Thus, we expected the difference in liver SUV to be − 80% with a standard deviation of ± 8%.

The noninferiority margin was difficult to access beforehand because it would depend on the difference between no treatment and WD standard of care treatment on hepatic SUV, since we had no literature to determine the margin (see supplementary material). It was however set to 15%.

Under these assumptions, the power calculation required only 6 participants in each group. However, allowing for dropouts, and because we were uncertain of both the noninferiority margin and the standard deviation, we decided to include 10 participants in each group.

### Statistics

The noninferiority method was used to examine if the test treatments were no less effective than the standard of care treatment (zinc acetate × 3 daily). The noninferiority margin was set to 25 percentage points (see supplementary material).

Data were collected in the REDCap electronic data capture tool hosted at Aarhus University and analyzed in STATA (StataCorp. 2017. Stata Statistical Software: Release 15. College Station, TX: StataCorp LLC) and GraphPad Prism (version 8.2.1 for macOS, GraphPad Software, San Diego, California USA)^32,33^.

Normality of data was controlled using Q-Q-plots, and one-way ANOVA and Student’s t-test were used for comparison of unpaired parametrically distributed data between four and two treatment groups, accordingly. Kruskal–Wallis H test and Wilcoxon rank-sum test were used for comparison of unpaired nonparametrically distributed data between four and two groups, respectively. Fisher’s exact test was applied for dichotomous outputs and Pearson’s, or Spearman’s test was used for testing the association of variables, depending on normality.

The intraindividual day-to-day variation was calculated as the coefficient of variation (CV) according to a standard method^34^.

Data are reported as mean values (standard deviation (SD)) unless otherwise specified. Analyses were performed on all completed participants and per protocol (PP). Both analyses regarding the main outcome are reported while the other outcomes and baseline characteristics are reported on all completed participants only.

### Ethics

The study was conducted as a randomized, non-blinded intervention study and was carried out in compliance with the Declaration of Helsinki II after approval from the regional Ethics Committee and the Danish Medicinal Agency. The trial was monitored by the regional unit for Good Clinical Practice and is registered in the European Union Drug Regulating Authorities Clinical Trials Database (EudraCT) as clinical trial number 2019-000905-57.

### Approvals and registration

The study was approved by The Central Denmark Region Committees on Health Research Ethics (1-10-72-41-19) and the Danish Medicines Agency (2019032179). The study was registered in the European Union Drug Regulating Authorities Clinical Trials Database (EudraCT) as clinical trial number 2019-000905-57. Results were posted on 10/07/2021.

### Access to data

D.E.M. and T.D.S. had access to all data and can vouch for the integrity of the data analyses.

## Supplementary Information

Supplementary Information.

## Data Availability

Deidentified participant data and the study protocol is available with the publication from the corresponding author on reasonable request.

## Abbreviations

^64^Cu: Copper-64
WD: Wilson’s disease
MT: Metallothionein
FDA: U.S. Food and Drug Administration
EMA: European Medicines Agency
PET: Positron emission tomography
CT: Computed tomography
SD: Standard deviation
MBq: Megabecquerel
CV: Coefficient of variation
SUV: Standard uptake value
EudraCT: European Union Drug Regulating Authorities Clinical Trials Database
INR: International standardized ratio
mL: Milliliters
NaCl: Sodium chloride
UltraHD: Ultra high definition
mm: Millimeters
VOI: Volume of interest
ALT: Alanine aminotransferase
CI: Confidence interval
NS: Non-significant
PP: Per-protocol
