# Supplementary material for: Effect of oral zinc regimens on human hepatic copper content: a randomized intervention study
Source: Sci Rep. 2022 Aug 29;12:14714. doi: 10.1038/s41598-022-18872-8 (PMC9424214; doi:10.1038/s41598-022-18872-8)
Supplement: Supplementary file 1 — Supplementary Information. [file 41598_2022_18872_MOESM1_ESM.pdf]

Statistics
The noninferiority analysis requires a margin (∂) which is usually determined by knowledge of treatment effect from meta-analyses. Since there are no data on zinc's effect on ^{64}Cu uptake, ∂ was determined by a two-sided 95% confidence interval around the estimated difference between the WD standard of care zinc regimen and no zinc regimen in combination with a clinical judgement about how much of ∂ should be preserved(1,2).
The estimated difference between standard of care zinc and no zinc in our study was -44.7 (95% CI: -75.4 to -14.6) percentage points (Supplementary material, Figure S1). We thus decided on a noninferiority margin (∂) of 25 percentage points, meaning that the test regimens were believed to be noninferior if the upper limit of the 95% confidence interval around the difference between means did not exceed 25 percentage points.

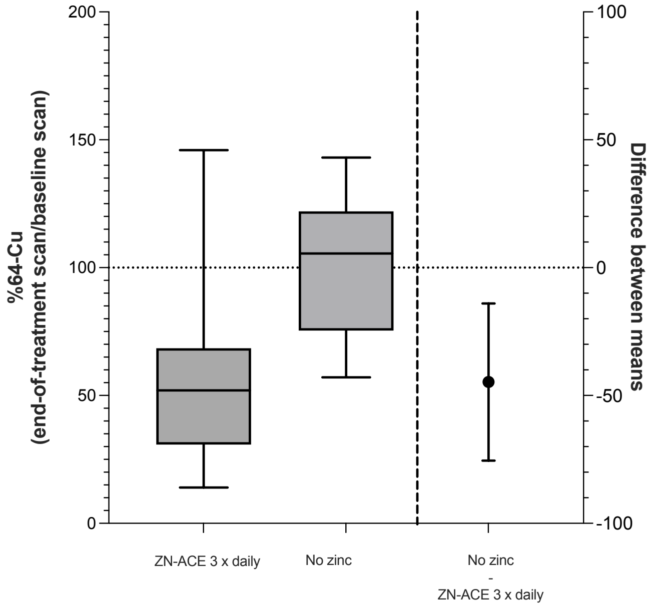

Figure S1. No zinc vs. standard of care zinc
Box plot of hepatic ^{64}Cu (mean hepatic SUV) as percentage on end-of-treatment scan compared to baseline scan
and
Difference between means (standard of care zinc vs. no zinc)

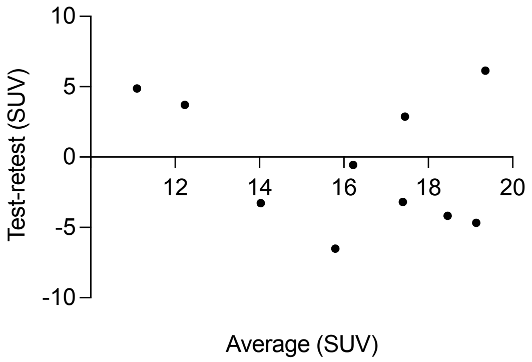

Figure S2. Bland-Altman plot
Test-retest (SUV)
Bias = -0.48

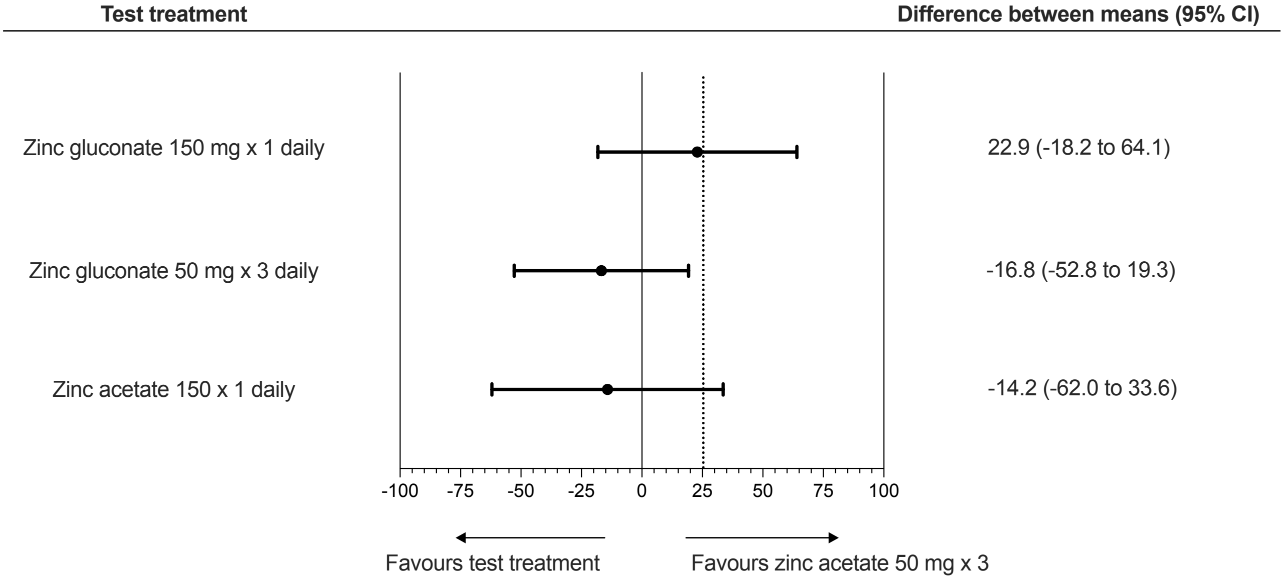

Figure S3. Difference between means (standard of care regimens vs. test regimens)
Per protocol analysis
Dotted line indicates noninferiority margin, ∂

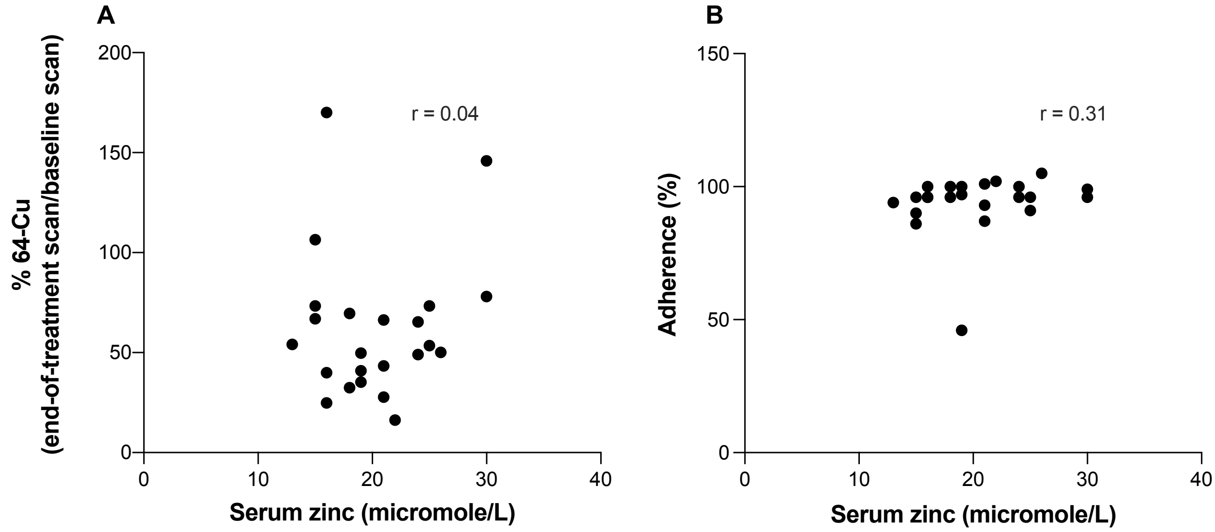

Figure S4. Correlations
A. Serum zinc and %^{64}Cu on end-of-treatment scan vs. baseline scan
B. Serum zinc and adherence

References

1. Rothmann M, Li N, Chen G, Chi GYH, Temple R, Tsou H-H. Design and analysis of non-inferiority mortality trials in oncology. Stat Med. 2003 Jan 30;22(2):239–64.

2. Research C for DE and. Non-Inferiority Clinical Trials [Internet]. U.S. Food and Drug Administration. FDA; 2020 [cited 2021 Aug 10]. Available from: https://www.fda.gov/regulatory-information/search-fda-guidance-documents/non-inferiority-clinical-trials
